# Metal–organic frameworks for H_2_ and CH_4_ storage: insights on the pore geometry–sorption energetics relationship

**DOI:** 10.1107/S2052252516019060

**Published:** 2017-02-10

**Authors:** Mohamed H. Alkordi, Youssef Belmabkhout, Amy Cairns, Mohamed Eddaoudi

**Affiliations:** a Division of Physical Sciences and Engineering, Advanced Membranes and Porous Materials Center, Functional Materials Design, Discovery and Development Research Group (FMD^3^), King Abdullah University of Science and Technology (KAUST), Thuwal 4700, Saudi Arabia

**Keywords:** tailored pore geometry, metal–organic frameworks, MOFs, hydrogen storage, dispersive interactions

## Abstract

This study aims to assess the possibility of improving H_2_ and CH_4_ binding affinity to the aromatic walls of a designed new Metal–Organic Framework (MOF) through simultaneous dispersive interactions. It is suggested here that desirable H_2_ and CH_4_ storage media at low pressures require narrow uniform pores associated with large surface area, a trade-off that is challenging to achieve.

## Abstract 2

Controlling the MOF pore geometry, size and functionality is of prime importance for the effective and selective gas adsorption. Gas adsorption studies on ultra-microporous MOFs were conducted to gain insight on the pore geometry–sorption energetics relationship.

## 1. Introduction   

Metal–Organic Frameworks (MOFs), an emerging class of functional solid-state materials, continue to receive wide scientific interest due to their potential applications in hydrogen storage, gas separation, carbon dioxide capture, enhanced catalysis and drug delivery (Eddaoudi *et al.*, 2001 ▸; Moulton & Zaworotko, 2001 ▸; Kitagawa *et al.*, 2004 ▸; Férey *et al.*, 2005 ▸; Horcajada *et al.*, 2006 ▸; Cho *et al.*, 2007 ▸). MOFs possess unique structural attributes including dual composition, crystallinity and a modular pore system (Rowsell & Yaghi, 2006 ▸; Belof *et al.*, 2007 ▸; Cho *et al.*, 2007 ▸; Dincă *et al.*, 2007 ▸; Hayashi *et al.*, 2007 ▸; Liu *et al.*, 2007 ▸; Alkordi *et al.*, 2008 ▸; Banerjee *et al.*, 2008 ▸; Llewellyn *et al.*, 2008 ▸; Nugent *et al.*, 2013 ▸; Belmabkhout *et al.*, 2014 ▸; Shekhah *et al.*, 2014 ▸). Markedly, these attributes are ideal for the assessment and the establishment of the requisite structure–function relationship toward the construction of made-to-order MOFs for a targeted application. In particular, porous MOFs are regarded as prospective adsorbents that can offer practical solutions to the enduring challenges pertaining to the safe storage and efficient use of H_2_ in mobile applications. Conceivably, MOFs are widely investigated for hydrogen storage due to the ability to control their pore system (functionality and volume) and subsequently impact the H_2_–MOF interactions and the total H_2_ uptake (Rowsell & Yaghi, 2005 ▸; Collins & Zhou, 2007 ▸; Lin *et al.*, 2007 ▸; Chen *et al.*, 2008 ▸; Dincă & Long, 2008 ▸; Nouar *et al.*, 2008 ▸; Kishan *et al.*, 2010 ▸; Zhou *et al.*, 2012 ▸). Our group, among others, continue to explore the modularity of MOFs in order to gain better insights on the structure–property relationship and subsequently construct a made-to-order MOF with the ideal gas–MOF interactions and suitable gas uptake for given gas separation/storage applications (Nugent *et al.*, 2013 ▸; Belmabkhout *et al.*, 2014 ▸; Shekhah *et al.*, 2014 ▸). Our study on the **soc**-MOF platform with the underlying square-octahedral (soc) topology (Belof *et al.*, 2007 ▸; Liu *et al.*, 2007 ▸; Alezi *et al.*, 2015 ▸; Cairns *et al.*, 2016 ▸) indicated that a made-to-order MOF suitable for hydrogen storage at relatively moderate pressures has to be highly porous (high surface area) and concomitantly encompass narrow pores (< 1 nm) and a high localized charged density (polarizable field charges). Notably, a large number of studies on CH_4_ and H_2_ storage by MOFs delineated the requirement of high surface area and high heat of adsorption for gas storage (Zhou *et al.*, 2012 ▸). Hydrogen interactions with metal complexes, clusters or ions, within the inorganic part of a given MOF, are dominated by electrostatic forces between the quadrupole moment of the hydrogen molecule and the inorganic complex. Specifically, such H_2_-MOF interactions’ strengths play a major role in determining the H_2_ uptake characteristics and hence are the subject of considerable theoretical and experimental investigations (Rowsell & Yaghi, 2005 ▸; Collins & Zhou, 2007 ▸; Hirscher & Panella, 2007 ▸; Lin *et al.*, 2007 ▸; Dincă & Long, 2008 ▸; Nouar *et al.*, 2008 ▸; Murray *et al.*, 2009 ▸; Zhou *et al.*, 2012 ▸). In particular, the weaker, dispersive interactions between H_2_ molecules and the organic linkers in MOFs, best represented by benzene ring moieties, have been theoretically investigated (Hübner *et al.*, 2004 ▸; Bhatia & Myers, 2006 ▸; Lochan & Head-Gordon, 2006 ▸; Düren *et al.*, 2009 ▸; Han *et al.*, 2009 ▸) and experimentally documented (Rosi *et al.*, 2003 ▸). Recent studies demonstrate that such interactions could, in principle, be enhanced through chemical modifications of the organic linkers, providing a prospective strategy for a material designer to fine-tune the organic building blocks and subsequently enhance H_2_ sorption characteristics of the MOF (Lochan & Head-Gordon, 2006 ▸). Nevertheless, to the best of our knowledge, no experimental synthetic studies have been published to address the potential to improve the H_2_ and CH_4_ binding affinity to the walls of MOFs through simultaneous dispersive interactions, acting additively, between the gas molecules and multiple aromatic rings placed at optimal interaction distance(s) within a specific geometry. Therefore, we opted to explore this approach separately, regardless of the degree of porosity, which could potentially pave the way for the rational design of MOF adsorbents as suitable and effective gas storage media. Computational studies revealed moderate binding affinities for the H_2_ molecule towards benzene and various aromatic rings (Hübner *et al.*, 2004 ▸; Bhatia & Myers, 2006 ▸; Lochan & Head-Gordon, 2006 ▸; Düren *et al.*, 2009 ▸; Han *et al.*, 2009 ▸). Such interactions are mostly dispersive and within the range of 3.4–4.0 kJ mol^−1^ for a H_2_ molecule interacting with the benzene ring of terephthalic acid (Lochan & Head-Gordon, 2006 ▸). The aforementioned binding enthalpy is remotely below the estimated and debated target for efficient H_2_ storage materials, range of 15–20 kJ mol^−1^ (room temperature at pressures up to 30 bar; Han *et al.*, 2009 ▸) or 21–32 kJ mol^−1^ (−20°C and pressure range 1–100 bar; Lochan & Head-Gordon, 2006 ▸). Herein, we set to investigate if such interactions could be additive and hence can lead to enhanced interactions between a H_2_ molecule and multiple aromatic rings in a tailored MOF adsorbent. As a test model, we envision a molecular square constructed of four benzene rings interacting simultaneously with a single H_2_ molecule, residing in the center of the square, as a potential model for a material with enhanced H_2_ binding affinity.

## 2. Experimental   

**Figure 1:**
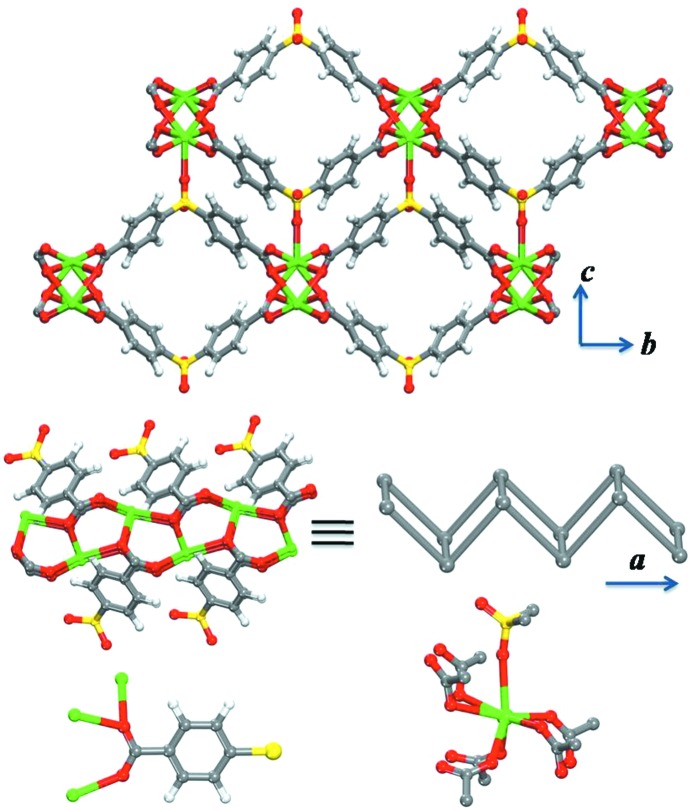
Crystal structure of **1** (top), the Pb—CO_2_ rod-shaped infinite SBU (middle), and the coordination mode of the carboxylic linker and the Pb(II) ion (below). Pb (green), C (gray), S (yellow), O (red), H (white).

The solvothermal reaction of Pb(NO_3_)_2_ and 4,4′-sulfonyldibenzoic acid in *N*,*N*-dimethylformamide (DMF) yields colorless crystals of **1** (Fig. 1 ▸). The as-synthesized compound was characterized and formulated by single-crystal X-ray diffraction studies as [Pb_2_(C_14_H_8_O_6_S)_2_]·DMF (**1**). The phase purity of **1** was confirmed by similarities between its calculated and experimental powder X-ray diffraction patterns (PXRD, supporting information).

A sample of **1** was activated for sorption studies by solvent exchange in acetonitrile, where complete removal of the DMF guest molecules was confirmed by IR spectroscopy, see the supporting information. The activated sample was found to be stable up to 400°C as confirmed by TGA studies, see the supporting information.

## 3. Results and discussion   

The crystal structure of **1** revealed square-like channels running through the *a*-axis. The distinctive shape of the ditopic ligand molecule (dihedral Ph—SO_2_—Ph angle of 103.93°) complemented by the coordination sphere around Pb(II) (CO_2_—Pb—CO_2_ dihedral angle of 78.73°) facilitated the construction of the MOF containing square-like channels (Table 1 ▸). In the crystal structure of **1**, infinite CO_2_—Pb(II) secondary building units (SBUs) (Rosi *et al.*, 2005 ▸) are observed and resulting from coordination of the carboxylate linkers in the bridging bis-bidentate mode to Pb(II) ions. Each carboxylate group is coordinated to three Pb(II) ions, enabling the formation of the CO_2_—Pb(II) infinite coordination chains, along the *a*-axis. Each Pb(II) ion is coordinated to six oxygen atoms from bridging carboxylate groups (O—Pb bond distances of 2.434–2.815 Å). Additionally, each Pb(II) ion is coordinated to an oxygen atom from the nearest sulfone group (O—Pb bond distance of 2.865 Å).

**Figure 2:**
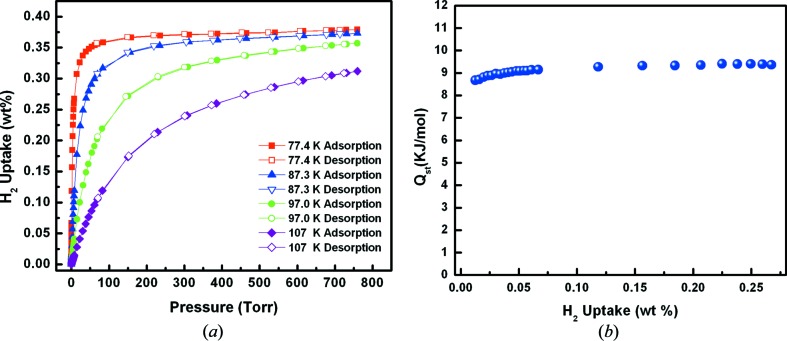
(*a*) Variable-temperature H_2_ adsorption isotherms and (*b*) *Q*_st_ of H_2_ adsorption in **1**.

**Figure 3:**
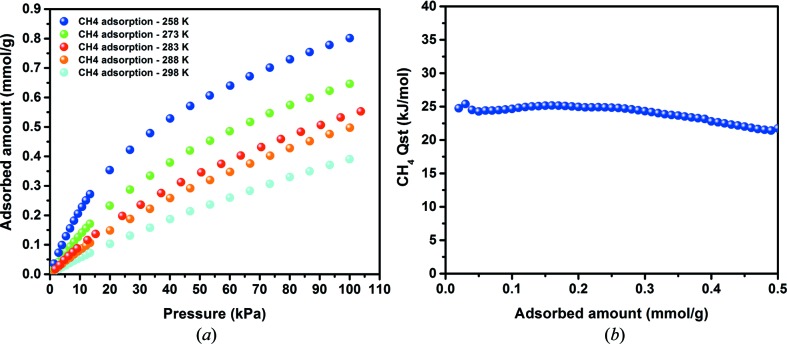
(*a*) Variable-temperature CH_4_ adsorption isotherms and (*b*) *Q*_st_ of CH_4_ adsorption in **1**.

The resultant connectivity of the Pb ions by the organic linkers facilitated the construction of parallelogram, square-like, shaped channels running along the *a*-axis, held together in the *bc*-plane through the sulfone–Pb coordination. Such orthogonal bridging interactions resulted in square-like, guest-accessible, channels in **1**. The surface area of **1** as probed by N_2_ and Ar at 77 K and 87 K (supporting information) were estimated to be 165 and 169 m^2^ g^−1^, respectively. The resulting square-like channels in **1** encompass a periodic array of aromatic rings with a relatively short interplanar distance between opposing rings (8.448 Å, centroid-to-centroid). Essentially, the periodically aligned aromatic rings delimiting the pore system dictate the pore aperture size and its maximum opening to be around **∼ 4 Å** (excluding the nearest van der Waals surfaces). These special structural features encountered in **1** (narrow one-dimensional channels aligned with a periodic array of aromatic rings) inspired us to explore and further investigate the potential effect(s) of the pore system (size, geometry and functionality) on the H_2_ interactions with the aromatic walls. Indeed, the observed H_2_ adsorption properties of the present MOF are remarkable and unique. Of special note are the observed H_2_ adsorption isotherms with a sharp steepness (type I isotherm shape particularly at 77 K), which, to the best of our knowledge, are scarce for physical adsorbents. Equally interesting is the observed steady H_2_ isosteric heat of adsorption (*Q*_st_) at 9 kJ mol^−1^ (Fig. 2 ▸). In fact, the H_2_ adsorption isotherms for **1** demonstrate rapid saturation at early dosing stages and nearly linear behaviour for heats of adsorption throughout the entire H_2_ adsorption loading, two highly desirable features for H_2_ storage applications. The sharp step in the H_2_ adsorption isotherm can be translated to sorption sites saturation by H_2_ molecules at moderate pressures. This behaviour could be attributed to the equal distribution of H_2_ sorption sites with uniform binding affinities within the framework, most probably on the surfaces of aromatic rings present in **1**. Interestingly, such uniform interactions were also observed for methane adsorption in **1** (Fig. 3 ▸), with a relatively high and steady *Q*_st_ of 25 kJ mol^−1^ over the entire loading range, thus confirming the interesting structural aspects of **1** for enhanced gas–solid adsorbent interactions.

**Figure 4:**
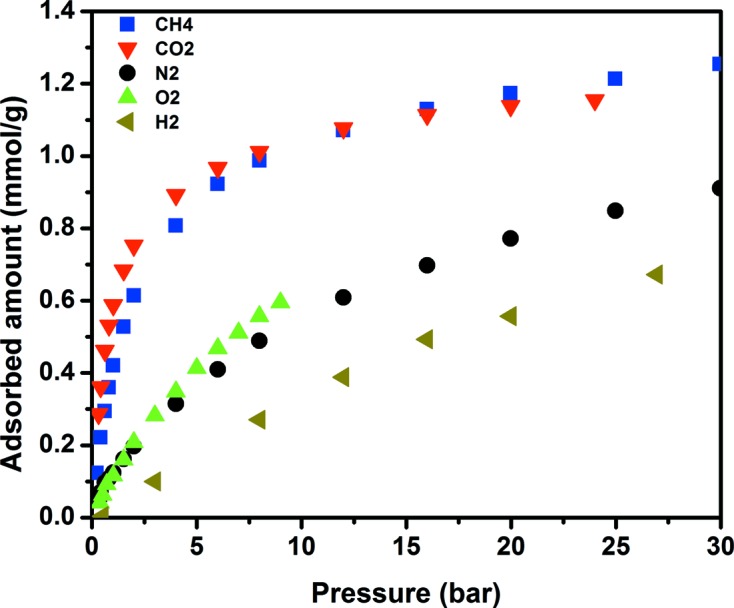
High-pressure sorption isotherms for different gases at 298 K in **1**.

Furthermore, high-pressure gas adsorption studies conducted on **1** revealed interesting adsorption behaviour of selected gases (Fig. 4 ▸). The recorded uptakes for O_2_ and N_2_ in **1** were comparable. This is in contrast to commonly observed preferential N_2_ uptake, compared with O_2_, in numerous examples of zeolites (Talu *et al.*, 1996 ▸; Hutson *et al.*, 1999 ▸; Agha *et al.*, 2005 ▸). In the case of most MOFs and zeolites, preferential N_2_ sorption is attributed to stronger interactions between N_2_ molecules and the material surface due to a larger quadrupole moment of N_2_. The interaction/adsorption sites in **1** are dominated by the periodic array of aromatic rings aligned in the one-dimensional channels, thus explaining the weak quadrupole interactions between the gas molecules and the framework and the subsequent comparable N_2_ and O_2_ uptakes. Similarly, the nature of the gas/framework interactions is also reflected in the observed comparable uptake of CH_4_ and CO_2_ (two distinct molecules with and without quadrupole moment) in **1** (Fig. 4 ▸), emphasizing the dominance of dispersive interactions between adsorbed gas molecules and the aromatic walls of the MOF porous material. It is noteworthy that porous materials having similar uptakes for CO_2_ and CH_4_ in a wide range of pressure is uncommon behaviour in adsorption on porous materials (Nugent *et al.*, 2013 ▸). Additionally, the adsorption of C_2_H_6_ and C_3+_ on **1** (supporting information) showed to some extent the same behaviour, uncommon for most MOF materials. The aforementioned results demonstrate that molecules with various degrees of high polarizabilities probe the surface of **1** in the same way. Specifically, the interaction potential of **1** with different molecules, having different chemical–physical properties like CO_2_, CH_4_, C_2_H_6_ and C_3+_, is governed mainly by dispersive (non-electrostatic) interactions.

## 4. Conclusions   

In conclusion, we present an unprecedented experimental illustration of uptake–energetics relationships for H_2_ and CH_4_ adsorption in a novel MOF with narrow one-dimensional channels aligned with a periodic array of aromatic rings. The newly synthesized material represents a model material for pinpointing the interplay between porosity/storage capacity and the strength of host–guest interactions. This approach supports the impact of narrow channels with a periodic array of aromatic rings, controlling the access to prospective larger pores within a targeted porous material, for the effective gas adsorption. The present results pave the way to additional experimental and theoretical investigations in order to further assess the extent of additive dispersive interactions in enhancing H_2_ and CH_4_ binding affinity in gas storage materials, in general, and MOFs, in particular. Noticeably, the distinct sharp step at relatively low pressures in the H_2_ adsorption isotherm at 77 K and the relatively high *Q*_st_ for CH_4_ storage was achieved despite the detrimental reduction in the MOF overall porosity. Conceivably, from the present study, the optimal combination of narrow pores/windows (< 1 nm diameter) with a suitable and uniform charge density in the pores (coordinatively unsaturated metal sites and polar functional groups) operating synergistically could play a significant role in promoting the storage of H_2_ and CH_4_ at moderate pressures and ambient temperatures.

## Supplementary Material

Crystal structure: contains datablock(s) kme002_0m. DOI: 10.1107/S2052252516019060/lq5001sup1.cif

Structure factors: contains datablock(s) kme002_0m. DOI: 10.1107/S2052252516019060/lq5001kme002_0msup2.fcf

Supporting adsorption and structural data. DOI: 10.1107/S2052252516019060/lq5001sup3.pdf

CCDC reference: 1528379

## Figures and Tables

**Table 1:** Selected geometric parameters (Å, °)

Pb1—O3^i^	2.434 (6)	Pb1—O4^i^	2.441 (6)
O3—Pb1—O4^i^	72.0 (2)	O2—S3—C6^ii^	108.1 (3)
O3^i^—Pb1—O4^i^	117.1 (2)	O1—S3—C6^ii^	107.6 (4)

Symmetry codes: (i) 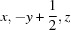; (ii) 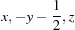.
