# Supplementary material for: Metal–organic frameworks for H2 and CH4 storage: insights on the pore geometry–sorption energetics relationship
Source: IUCrJ. 2017 Feb 10;4(Pt 2):131–5. doi: 10.1107/S2052252516019060 (PMC5330523; doi:10.1107/S2052252516019060)
Supplement: Supplementary file 3 [file m-04-00131-sup3.pdf]

IUCrJ

Volume 4 (2017)

Supporting information for article:

Metal-Organic Frameworks for H2 and CH4 Storage: Insights on the Pore Geometry-Sorption Energetics Relationship

Mohamed H. Alkordi, Youssef Belmabkhout, Amy Cairns and Mohamed Eddaoudi

Supporting Information

Metal-Organic Frameworks for H_2 and CH_4 Storage: Insights on the Pore Geometry-Sorption Energetics Relationship

Mohamed H. Alkordi,[a] Youssef Belmabkhout,[a] Amy J. Cairns,[a] and Mohamed Eddaoudi*[a]

[a] *King Abdullah University of Science and Technology(KAUST), Division of Physical Sciences and Engineering, Advanced Membranes and Porous Materials Center, Functional Materials Design, Discovery and Development Research Group (FMD[3]), , Thuwal, 4700, Saudi Arabia*

Table of Contents

1) Materials and Methods: 2

2) Synthesis: 3

3) PXRD: 3

4) Gas Sorption Isotherms: 4

5) FT-IR spectra: 6

6) TGA: 7

7) Table S1. Experimental details for1 8

8) References 9

1) Materials and Methods:

All chemicals were of commercial grade purity used as received without further purification.Fourier-transform (FT-IR) spectra (4000-400 cm-1) were collected by using a Nicolet AVATAR 320FT-IR. X-ray Powder Diffraction (XRPD) measurements were carried on a PANalytical X`PERT diffractometer with Cu source (45kV, 40mA, CuKα λ = 1.5418 Å) at room temperature. Gas sorption studies were performed on a Quantachrome Autosorb-1 equipped with Oxford MK2 Cryostat. Single-crystal data was collected using Bruker APEX II QUAZAR with IμS™ X-ray Source Cu Kα radiation (λ = 1.5418 Å), a 30 W micro focus sealed tube with high brilliance and high-performance focusing Quazar™ multilayer optics, the latest type of Montel mirror and APEXII CCD detector. Indexing was performed using SMART v5.625. [1] Frames were integrated with SaintPlus 6.28A [2] software package. Absorption correction was performed by multi-scan method implemented in SADABS.[3] Crystal structures were solved using SHELXS-97 and refined using SHELXL-97 contained in SHELXTL v6.10 and WinGX v1.70.01 programs packages. [4] All non-disordered non-hydrogen atoms were refined with anisotropic displacement parameters. All H-atoms bonded to carbon atoms were placed in geometrically optimized positions. The highly disordered with low occupancy electron density residuals inside the channels of the material could not be modeled adequately and the SQUEEZE treatment included in PLATON[5] software was used.

2) Synthesis:

Synthesis of **1:** Solvothermal reaction of equimolar amounts (0.1 mmol, 0.033g) of $Pb(NO_3)_2$ and 4,4'-sulfonyldibenzoic acid (0.1 mmol, 0.031g) in *N,N'*-dimethylformamide (DMF) (1 mL) at 115°C for 12 h resulted in colorless rectangular crystals of **1** formulated as $[Pb(C_{14}SO_6H_8)]_2$(0.0514 g, quantitatively) using single crystal X–ray diffraction study.

Solvent exchange of **1:** The as-synthesized crystalline material is washed several times with acetonitrile, in which it is soaked for 20 days at room temperature, where the solvent was frequently replaced.The solvent-exchanged sample is then placed under dynamic vacuum at room temperature for 26h, followed by gradual heating to 120°C for 12h before conducting the various gas sorption measurements reported herein.

3) PXRD:

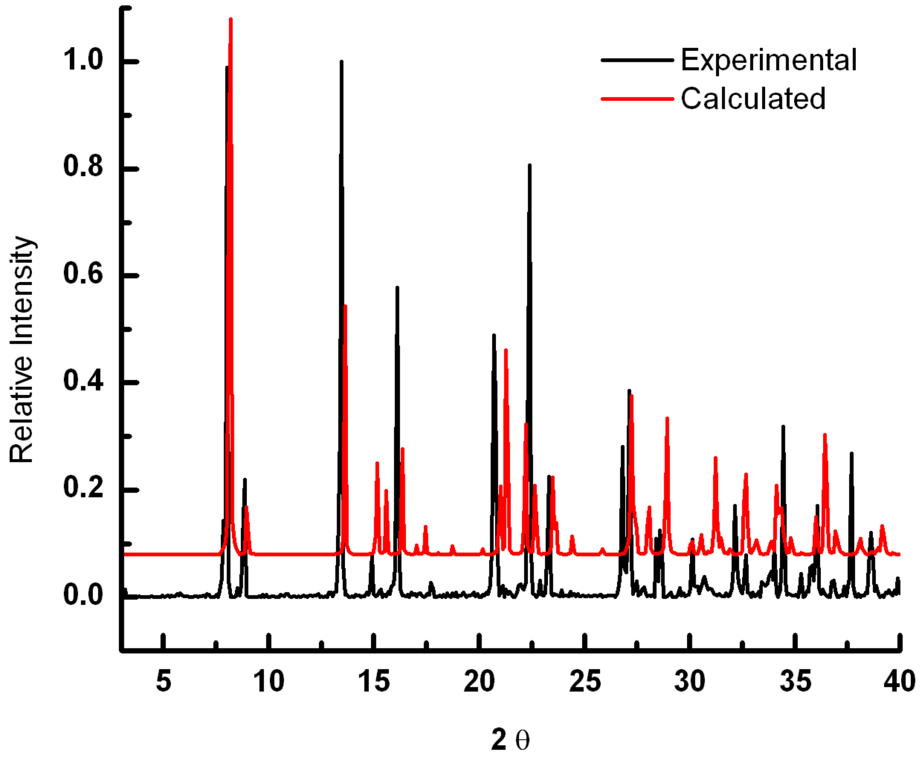

Figure S1. Powder X-ray diffraction (PXRD) patterns for the as-synthesized **1**(black line) and the calculated pattern (red line).

4) Gas Sorption Isotherms:

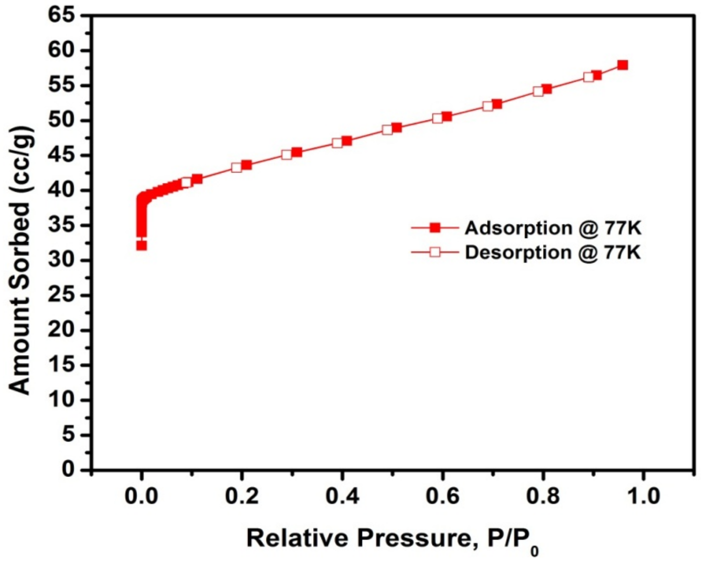

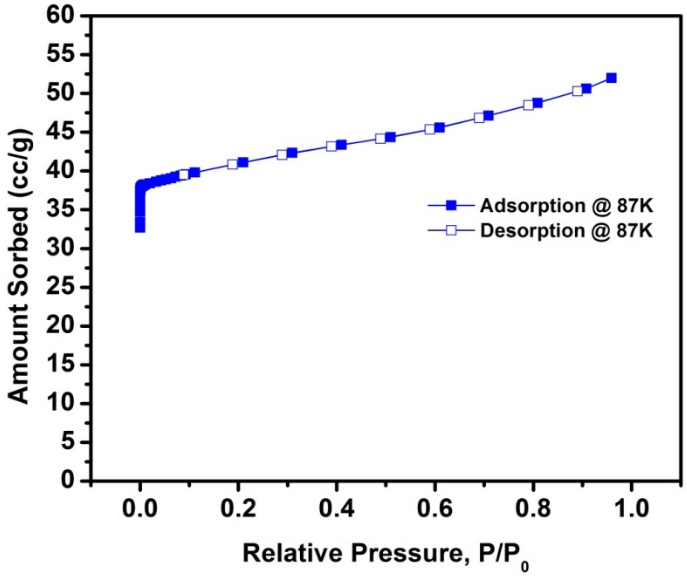

Figure S2. (Top) N_2sorption isotherm for **1**, 77 K and (below) Argon sorption isotherm for **1**, 87 K.

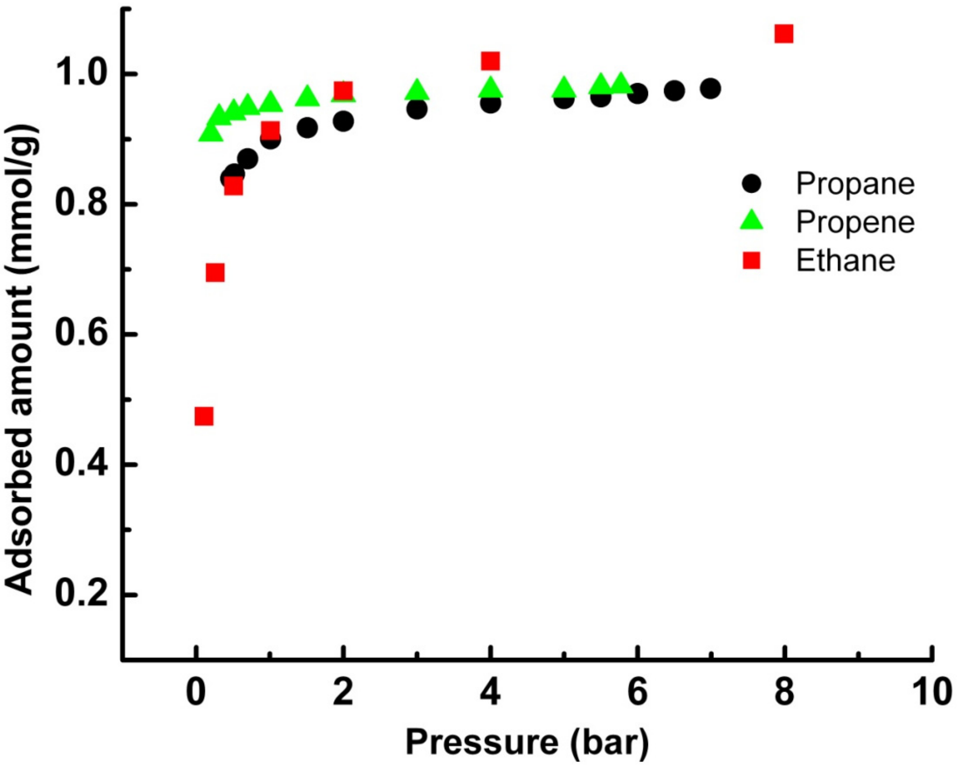

Figure S3: Gas sorption isotherms for Propane, Propene, and Ethane in **1**

5) FT-IR spectra:

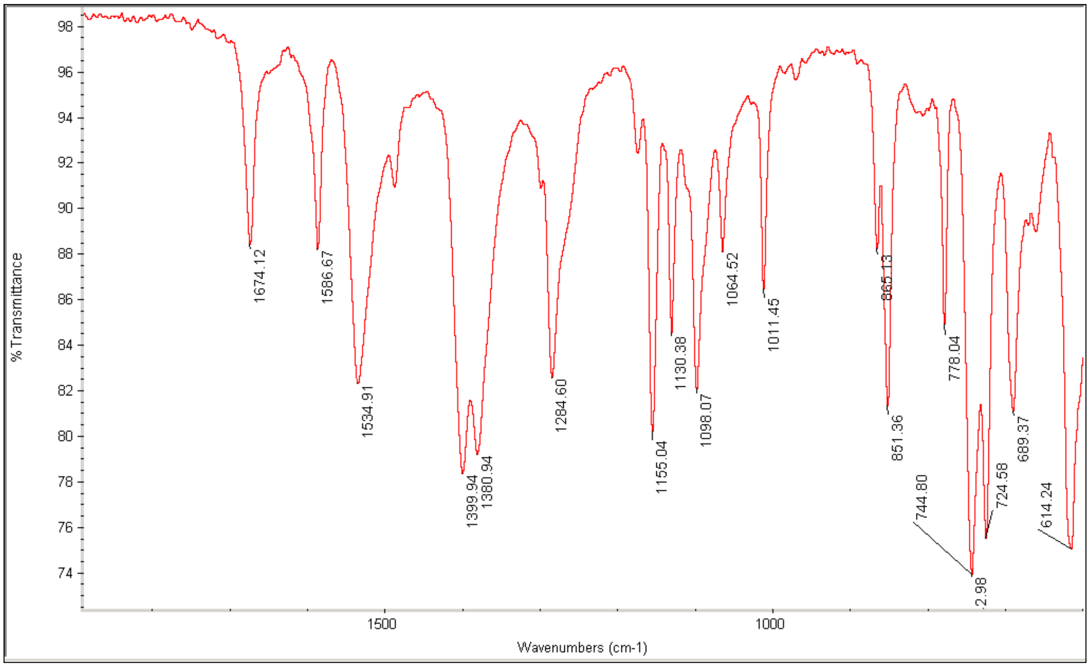

Figure S4. FT-IR spectrum of **1**

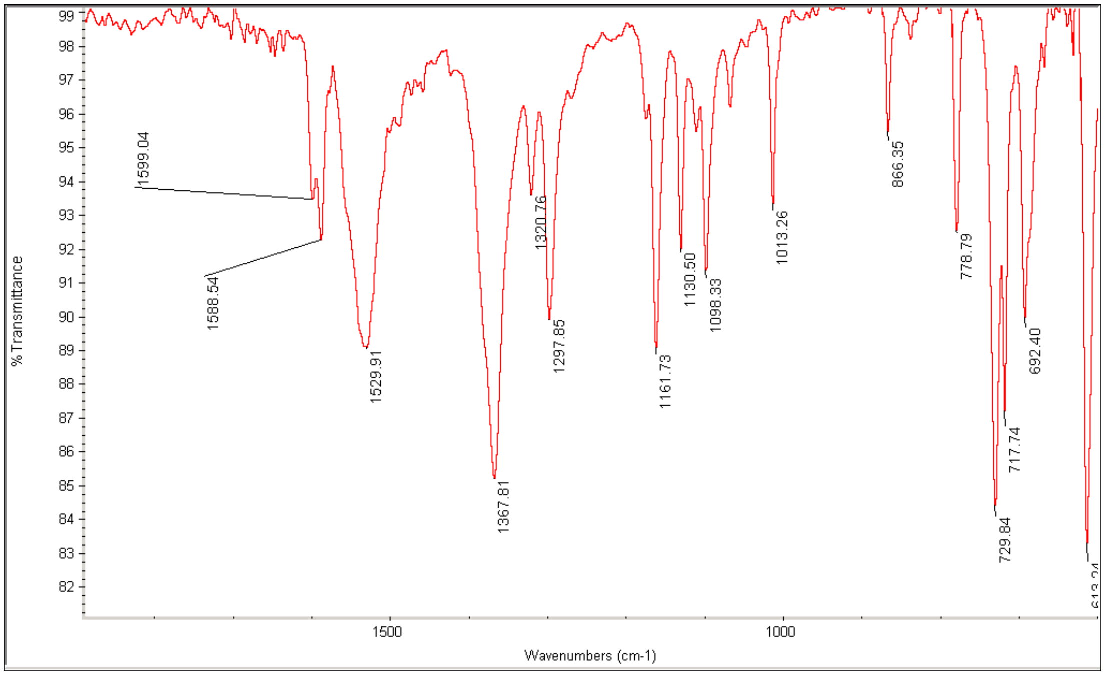

Figure S5. FT-IR spectrum of **1** after solvent wash and exchange of DMF with acetonitrile (loss of absorption band at 1674 cm^{-1}).

6) TGA:

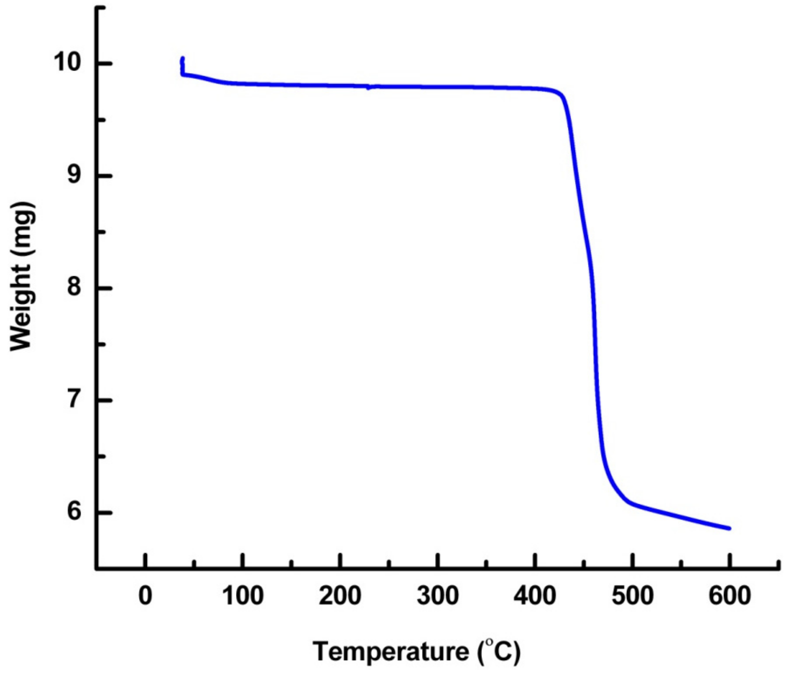

Figure S6. TGA for **1** after solvent wash and exchange in acetonitrile.

7) Table S1. Experimental details for1

	1
Crystal data	
Chemical formula	$C_{14}H_8O_6PbS$
M_r	511.45
Crystal system, space group	Orthorhombic, *Pnma*
Temperature (K)	100
a, *b*, *c* (Å)	5.8740 (4), 13.0820 (7), 19.2970 (12)
V (Å^3)	1482.85 (16)
Z	4
Radiation type	Cu *K*α
μ (mm^{-1})	23.70
Crystal size (mm)	0.10 × 0.05 × 0.05
Data collection	
Absorption correction	Multi-scan
T_{min}, T_{max}	0.200, 0.384
No. of measured, independent and observed [$I > 2\sigma(I)$] reflections	3462, 1232, 1184
R_{int}	0.036
$(\sin \theta/\lambda)_{max}$ (Å^{-1})	0.587
Refinement	
$R[F^2 > 2\sigma(F^2)]$, $wR(F^2)$, S	0.039, 0.101, 1.15
No. of reflections	1232
No. of parameters	106
No. of restraints	0
H-atom treatment	H-atom parameters constrained
	$w = 1/[\sigma^2(F_o^2) + (0.0363P)^2 + 22.3978P]$ where $P = (F_o^2 + 2F_c^2)/3$
$\Delta\rho_{max}$, $\Delta\rho_{min}$ (e Å^{-3})	2.62, −1.36

Computer programs: *SHELXS97* (Sheldrick, 2008), *SHELXL97* (Sheldrick, 2008).

8) References

1. Bruker-AXS (2001). SMART-V5.625. Data Collection Software. Madison, Wisconsin, USA.
2. Bruker-AXS (2001). SAINT-V6.28A. Data Reduction Software. Madison, Wisconsin, USA.
3. Sheldrick, G. M. (1996). SADABS. Program for Empirical Absorption Correction. University of Gottingen, Germany.
4. (a) Sheldrick, G. M. SHELXTL, v. 6.10; Bruker-AXS Madison, Wisconsin, USA. 2000. (b) Farrugia L. *J. Appl. Cryst.* **1999**, *32*, 837-838. (c) Sheldrick, G.M. *Acta Cryst*. **2008**, *A64*, 112-122. (d) Sheldrick, G.M. *Acta Cryst.***1990**, *A46*, 467-473.
5. (a) Spek, A.L. *J. Appl. Cryst.* **2003**, 36, 7-13. (b) Spek, A.L. *Acta Cryst.* **2009**, D65, 148-155.
